# Potential Ameliorative Effect of *Zosima absinthifolia* on Diminished Ovarian Reserve in Rats

**DOI:** 10.5812/ijpr-172634

**Published:** 2026-09-20

**Authors:** Leila Safarian, Homa Hajimehdipoor, Mojgan Tansaz

**Affiliations:** 1 Traditional Medicine and Materia Medica Research Center, Shahid Beheshti University of Medical Sciences Tehran Iran; 2 Department of Traditional Pharmacy, School of Traditional Medicine, Shahid Beheshti University of Medical Sciences Tehran Iran; 3 Department of Traditional Medicine, School of Traditional Medicine, Shahid Beheshti University of Medical Sciences Tehran Iran

**Keywords:** Anti-Müllerian Hormone (AMH), Cyclophosphamide, Diminished Ovarian Reserve (DOR), Persian Medicine, *Zosima absinthifolia*

## Abstract

**Background:**

Diminished ovarian reserve (DOR), a leading cause of female infertility, has been increasing worldwide and currently lacks an effective and specific treatment. *Zosima absinthifolia* has traditionally been used in Persian medicine to treat menstrual disorders and enhance libido.

**Objectives:**

To investigate the protective effects of *Z. absinthifolia* extract against cyclophosphamide-induced DOR in a rat model.

**Methods:**

*Zosima absinthifolia* was extracted with 80% methanol and dried. Thirty female rats were randomly allocated to five groups: sham, DOR, and three extract-treated groups (12.5, 25, and 50 mg/kg). DOR was induced by a single intraperitoneal injection of cyclophosphamide (45 mg/kg). Fourteen days later, the rats received the extract orally for 28 days. In an initial exploratory dose-screening phase, serum anti-Müllerian hormone (AMH) levels were used to identify the most biologically active dose. Based on these findings, an optimal dose was selected for subsequent hormonal and histopathological evaluations.

**Results:**

Cyclophosphamide significantly decreased serum AMH and estradiol (E_2_) levels and increased follicle-stimulating hormone (FSH) levels compared with those in the sham group. Treatment with *Z. absinthifolia* (12.5 mg/kg) significantly increased AMH and E_2_ levels and reduced FSH levels compared with those in the DOR group. Although treatment increased the numbers of primordial, primary, and antral follicles, these differences were not statistically significant. Similarly, no significant differences were observed in the numbers of Graafian follicles, cystic follicles, or corpora lutea among the groups.

**Conclusions:**

These findings provide preliminary evidence that *Z. absinthifolia* extract may exert protective effects in a rat model of cyclophosphamide-induced DOR. However, further studies evaluating toxicity, reproductive safety, dose-response relationships, and conducting controlled clinical trials are required before its potential clinical application can be considered.

## 1. Background

Fertility plays an important role in the continuation of generations. With the increasing prevalence of infertility in modern society, research has increasingly focused on identifying its causes; among these, diminished ovarian reserve (DOR) has become one of the most common challenges in fertility care and clinical reproductive medicine (1). Diminished ovarian reserve refers to a reduction in both the quantity and quality of ovarian oocytes, accompanied by decreased anti-Müllerian hormone (AMH), an important biomarker of ovarian reserve, a reduced number of antral follicles, and increased follicle-stimulating hormone (FSH) levels (2, 3). Diminished ovarian reserve can develop due to a wide range of risk factors, with advanced age (> 35 years) being a primary contributor. Additional risk factors include conditions that directly damage the ovaries, such as endometriosis, pelvic infections, or pelvic tuberculosis; previous ovarian surgery; a family history of early menopause; certain genetic predispositions; exposure to chemotherapy and pelvic radiation therapy; autoimmune disorders; lifestyle factors such as smoking; and various environmental exposures (3). Because the mechanisms and clinical presentations of DOR are complex, treatment remains challenging, and there is still no universally accepted safe and effective therapy for DOR (4). Several interventions may improve fertility outcomes, including platelet-rich plasma (PRP) (5), dehydroepiandrosterone (DHEA) supplementation (6), clomiphene citrate, melatonin, letrozole (3), lifestyle modifications (7), and assisted reproductive technologies (ART) (8). However, prolonged use of some of these agents may increase the risk of conditions such as endometrial and breast cancers (9). Given the absence of a specific therapy for DOR, identifying optimal treatments with minimal adverse effects remains an important research priority. In this context, Persian medicine (PM; Iranian traditional medicine), with its long history, represents a valuable source of medicinal plants for drug discovery.

*Zosima absinthifolia* (Vent.) Link (Apiaceae), known as “Anjedan-e roomi” in PM, is a perennial herbaceous plant that grows wild on mountainous and stony slopes in Central Asia and Iran (10). The plant is rich in biologically active compounds, including coumarins, furocoumarins, pyranocoumarins, flavonoids, monoterpenes, sesquiterpenes, and essential oils (11). Constituents identified in various plant parts include zosimin, deltoin (coumarin lactones), angelicin, umbelliferone, and several flavonoids and chalcones (12, 13). In PM, its fruit is used for gastrointestinal disorders such as indigestion, gastric flatulence, and stomachache, as well as to enhance menstruation and libido.

Animal models of DOR play a crucial role in evaluating the efficacy and pharmacological functions of bioactive compounds. Available DOR models include chemical toxin models, such as 4-vinylcyclohexene diepoxide (14); chemotherapy-induced models, such as those using cyclophosphamide (15); stress-related paradigms, such as chronic unpredictable stress (16); autoimmune models using zona pellucida 3 fragments (17); and natural aging models (18). Chemotherapy-induced models are simple, reproducible, and reliably mimic the clinical features of DOR, with drug-reversible changes (19). Cyclophosphamide-induced ovarian injury is one of the most widely accepted experimental models. Cyclophosphamide causes depletion of the ovarian follicular pool through DNA damage, apoptosis of granulosa cells and oocytes, oxidative stress, and inflammatory responses, resulting in reduced serum AMH, elevated FSH, and impaired ovarian function (15). These pathological and endocrine alterations resemble the principal characteristics of chemotherapy-induced DOR in humans and therefore provide a useful preclinical model for evaluating potential ovarian-protective agents.

Although no direct mechanistic evidence has previously linked *Z. absinthifolia* to the treatment of DOR, its long-standing use in PM for female reproductive disorders, together with its reported antioxidant and anti-inflammatory phytochemical constituents, such as phenolics and coumarins (20, 21), provides a rationale for evaluating its effects on cyclophosphamide-induced DOR, which is partly associated with oxidative stress and inflammation in the ovaries.

## 2. Objectives

This study aimed to evaluate the effects of the total extract of *Z. absinthifolia* fruits on DOR-related complications induced by cyclophosphamide in an animal model.

## 3. Methods

### 3.1. Ethical Considerations

All experimental procedures were approved by the Animal Ethics Committee of Shahid Beheshti University of Medical Sciences (No. IR.SBMU.AEC.1404.010). The study was designed and reported in accordance with the ARRIVE 2.0 guidelines (22). Animals were monitored daily for health and welfare, and all procedures were performed by trained personnel.

### 3.2. Chemicals and Instruments

Cyclophosphamide (Endoxan) was purchased from Baxter Oncology Company, Germany. The warfarin standard was obtained from USP, Sweden. Eosin, hematoxylin, and other chemicals were obtained from Sigma-Aldrich (St Louis, MO, USA), and lead (II) acetate trihydrate was obtained from Merck, Germany. Rat hormone assay kits were as follows: rat AMH ELISA kit, cat. No. EA0083Ra; rat estradiol (E_2_) kit, cat. No. KGE014, R&D, USA; and rat FSH kit, cat. No. Y2310012, Biotime, China. Analytical UV spectrophotometry was performed using a PerkinElmer Lambda 25 UV/VIS spectrometer.

### 3.3. Plant Material

*Zosima absinthifolia* fruits were purchased from an herbal market in Tehran, Iran, and identified by a botanist at the Herbarium of the Traditional Medicine and Materia Medica Research Center (TMRC), Shahid Beheshti University of Medical Sciences, Tehran, Iran, under code HMS-587. Quality-control tests were performed on the *Z. absinthifolia* fruits. The total extract of *Z. absinthifolia* fruits was prepared by maceration with shaking, using 80% methanol as the solvent for 24 h at a plant-to-solvent ratio of 1:10 (w/v). The extract was concentrated using a rotary evaporator at 40 °C and subsequently dried using a freeze dryer.

### 3.4. Thin-Layer Chromatography

Thin-layer chromatography (TLC) was used to identify different constituents of the plant. Based on related plants, coumarin compounds, flavonoids, and essential-oil constituents were assessed.

#### 3.4.1. Identification of Coumarin Compounds

The methanolic plant extract was prepared by maceration at a plant-to-solvent ratio of 1:10 for 24 h. The stationary phase was silica gel, the mobile phase was toluene:diethyl ether saturated with 10% acetic acid (1:1), and alcoholic KOH was used as the reagent.

#### 3.4.2. Identification of Flavonoid Compounds

The stationary phase was silica gel, the mobile phase was ethyl acetate:formic acid:glacial acetic acid:water (100:11:11:26), and Natural Product (NP)/PEG 4000 was used as the reagent.

#### 3.4.3. Identification of Essential-Oil Compounds

The stationary phase was silica gel, the mobile phase was toluene:ethyl acetate (93:7), and anisaldehyde/sulfuric acid with heating was used as the reagent.

### 3.5. Quantification of Total Coumarins as Warfarin in the *Z. absinthifolia* Extract

The purpose of this assay was to standardize the extract rather than to identify or quantify individual coumarin constituents. Therefore, the total coumarin content of the extract was determined by UV spectrophotometry using warfarin as the reference standard to construct the calibration curve. Results were expressed as the percentage of warfarin equivalents in the dry extract.

#### 3.5.1. Sample and Standard Preparation

For the quantitative determination of total coumarins, 0.1 N NaOH was used as the blank solution, and lead acetate was used as the reagent (0.5 g of lead acetate was dissolved in 10 mL of hot water). Different concentrations of warfarin (10 - 20 ppm) were prepared in the blank solution to construct a standard calibration curve. For sample preparation, 7.5 mg/mL of *Z. absinthifolia* dry extract was prepared in 80% methanol.

#### 3.5.2. Quantification Method

A 0.5-mL aliquot of the prepared sample solution was mixed with 0.5 mL of lead acetate solution, diluted to 10 mL with water, and shaken for 5 min. Lead acetate precipitated all sample components except coumarins, yielding a cloudy yellow solution. After centrifugation at 2500 rpm for 10 min, 2 mL of the supernatant was diluted to 10 mL with the blank solution and filtered through a 0.45-µm filter. Sample absorbance was measured at 280 nm. The total coumarin concentration was calculated using the warfarin calibration curve (23, 24).

### 3.6. Animal Study

#### 3.6.1. Animals

Thirty female Sprague Dawley rats weighing 160 - 210 g and aged 6 - 8 weeks were obtained from the TMRC. The animals were maintained under controlled conditions at 22 ± 2 °C, with a 12-h light/dark cycle and relative humidity of 45% - 65%. The rats had unrestricted access to food and water and received a standard diet. The study was conducted between October 2024 and January 2025 in the laboratories of the TMRC, Shahid Beheshti University of Medical Sciences, Tehran, Iran. Hormonal analyses were performed at Soren Laboratory, Tehran, Iran, and histopathological evaluations were performed in the Department of Pathology, Imam Khomeini Hospital, Tehran, Iran.

#### 3.6.2. Cyclophosphamide-Induced DOR Model and Treatments

The doses of *Z. absinthifolia* extract were selected based on the traditional daily human dose of 3 g/day described in Persian medicine textbooks. The human dose was converted to the equivalent rat dose using the standard body-surface-area normalization method (25). Considering the extraction yield of 8.33%, the equivalent extract dose was calculated to be approximately 25 mg/kg, which was selected as the intermediate dose. Half (12.5 mg/kg) and twice (50 mg/kg) this dose were selected as the low- and high-dose groups, respectively, to explore the optimal dose.

Female Sprague Dawley rats were screened and synchronized according to the estrous cycle using vaginal smear examinations, and 30 rats were then randomly allocated to the experimental groups (sham, DOR, and treatment groups) using a random-number table. The sample size (n = 6 animals per group) was selected based on previous studies using similar experimental models (26) and in accordance with the Reduction principle of the 3Rs (27). No formal a priori sample-size calculation was performed because this study was designed as an exploratory preclinical investigation. No animals were excluded after randomization.

The study was designed as an exploratory preclinical investigation consisting of 2 sequential phases. In the initial dose-screening phase, 3 doses of *Z. absinthifolia* extract (12.5, 25, and 50 mg/kg) were evaluated using serum AMH as the primary screening biomarker to identify the biologically active dose. Based on the screening results, the 12.5 mg/kg dose was selected in the second phase for subsequent comprehensive hormonal and histopathological evaluations.

Based on pilot data, cyclophosphamide at 45 mg/kg was used to establish the DOR model in rats (AMH = 4.89 ng/mL ± 2.34 vs 12.35 ng/mL ± 2.80; P = 0.0132). All groups except the sham group received a single intraperitoneal injection of cyclophosphamide dissolved in normal saline, whereas rats in the sham group received the same volume of normal saline. Fourteen days after cyclophosphamide administration, *Z. absinthifolia* extract was administered orally by gavage for 28 days. Rats in the sham and DOR groups received the same volume of deionized water. After 28 days of treatment, blood was obtained by cardiac puncture, and the ovaries were removed from rats euthanized with CO_2_ for further experiments (28, 29).

#### 3.6.3. Hormone Measurement

Serum AMH, FSH, and E_2_ levels were assessed using specific ELISA kits. All procedures were performed according to the manufacturers' instructions, and hormone levels were determined using a calibration curve for each assay. A small number of individual hormone measurements were excluded before statistical analysis because of technical assay failure. Investigators performing the hormone measurements were blinded to experimental group allocation throughout sample analysis.

#### 3.6.4. Histopathological Analysis

The ovaries were excised, weighed, fixed in 10% neutral-buffered formalin, embedded in paraffin, and sectioned at a thickness of 5 µm. Ovarian sections from the different groups were stained with hematoxylin and eosin (H&E). Both ovaries from 5 rats in each group and 3 representative serial sections from each ovary, with a 20-µm trimming interval, were selected for histopathological evaluation.

Assessment was performed by an experienced histopathologist who was blinded to experimental group allocation.

Follicles were systematically enumerated in each section using the microscope stage along the X and Y axes. Classification criteria were as follows: primordial follicles, an oocyte with a single layer of flattened cells; primary follicles, an oocyte with a single cuboidal cell layer; antral follicles, an oocyte with an antral cavity filled with follicular fluid; Graafian follicles, a very large follicle containing a clearly identifiable eccentrically positioned oocyte surrounded by a well-developed cumulus oophorus and prominent granulosa/theca layers; and corpus luteum, a structure clearly distinct from an intact follicle and showing morphological atresia. To avoid double counting, only follicles containing a visible oocyte nucleus were included. Mean values obtained from both ovaries were used for statistical analysis. Ovarian inflammation was identified by vascular congestion, edema, hemorrhage, leukocyte infiltration, and follicular degeneration (30, 31). The ovarian index was calculated for each rat as follows (29):

Ovarian index (%) = [ovarian weight (g) / body weight (g)] × 100

#### 3.6.5. Statistical Analysis

All data are presented as mean ± standard deviation (SD) and were analyzed using GraphPad Prism 9.0. The individual rat was considered the experimental unit for all statistical analyses. For histological assessments, the average values obtained from both ovaries were used for each animal. After assessment of data normality and homogeneity of variance, comparisons among multiple groups were performed using one-way analysis of variance (ANOVA), followed by Tukey's multiple-comparison test. Statistical significance was defined as P < 0.05. Exact P values are reported where available.

## 4. Results

Because no monograph is available for *Z. absinthifolia*, a general protocol was followed. Table 1 presents the quality-control results for *Z. absinthifolia* fruits.

**Table 1.** Physicochemical Analysis of *Zosima absinthifolia* Fruits

Tests	Result
**Description**	Oval compressed concave fruit with pungent acrid taste and aromatic scent
**Foreign matter**	5.22 ± 0.05%
**Total ash**	10.00 ± 0.26%
**Essential oil**	4.00 ± 0.10%
**Loss on drying**	1.43 ± 0.06%
**Methanol 80% extractive**	8.33 ± 0.22%

In thin-layer chromatography of the plant methanolic extract, coumarin compounds were observed as intense blue and blue-green zones at R_f_ = 0.29 and R_f_ = 0.52. Phenolic and flavonoid compounds were separated as distinct yellow-orange spots at R_f_ = 0.51 and blue spots at R_f_ = 0.41. Essential-oil constituents appeared as distinct green (R_f_ = 0.27), purple (R_f_ = 0.39), and pink (R_f_ = 0.41) spots.

According to the warfarin calibration curve (Y = 0.0228X + 0.0126), the total coumarin content of the *Z. absinthifolia* extract, expressed as warfarin equivalents, was 20.71 ± 0.75%.

As shown in Figure 1, the ovarian index in the DOR group was significantly lower than that in the sham group (P = 0.0296). Treatment with *Z. absinthifolia* at 12.5 mg/kg significantly improved the ovarian index compared with that in the DOR group (P = 0.0317). No significant changes in ovarian index were observed in the other treatment groups.

**Figure 1.**
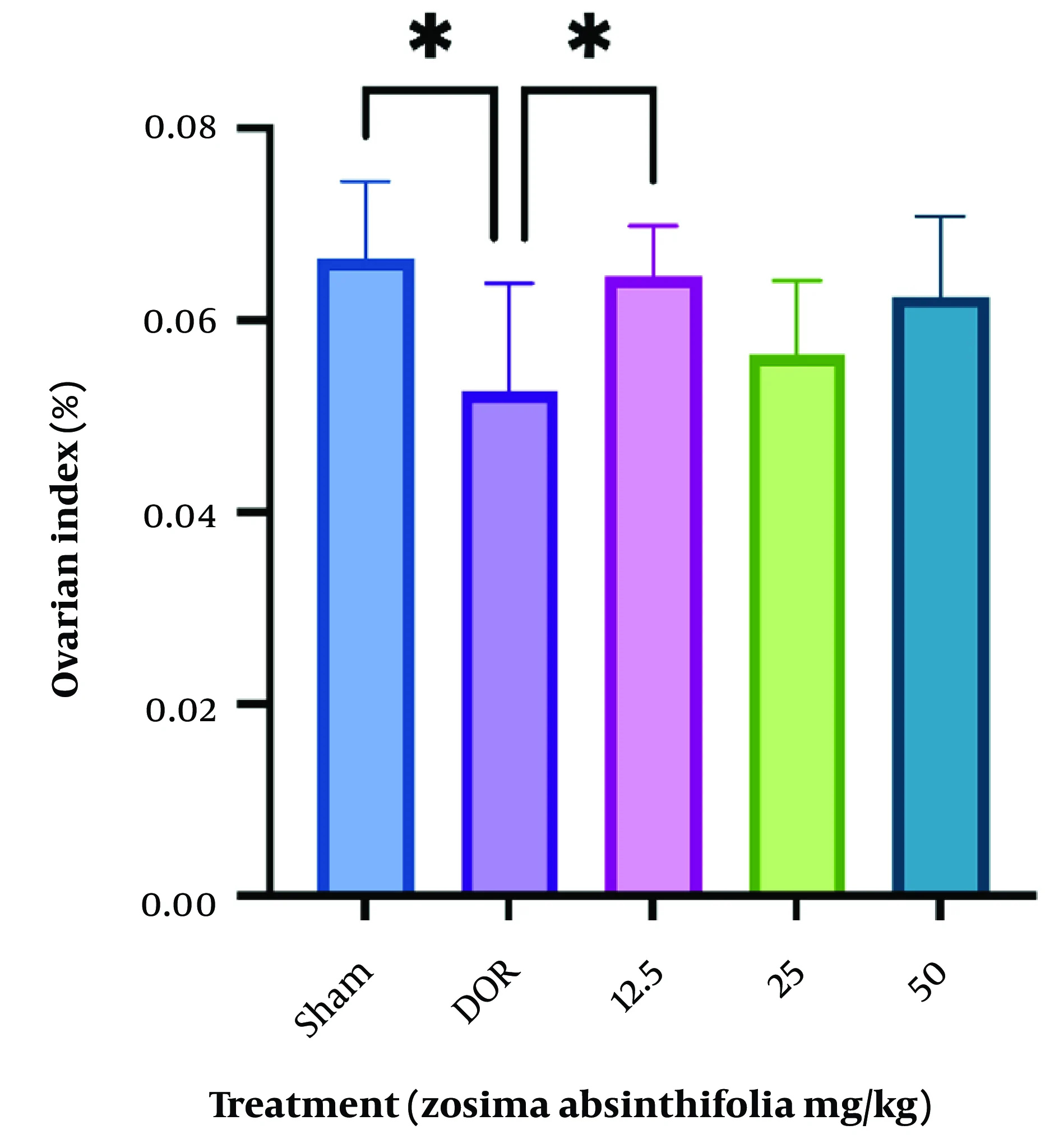
Ovarian index in rats with diminished ovarian reserve (DOR) after treatment with *Zosima absinthifolia* extract (12.5, 25, or 50 mg/kg) for 28 days. *P < 0.05. Values are expressed as mean ± standard deviation (SD) (n = 6).

To identify the biologically active dose of *Z. absinthifolia*, an exploratory dose-screening experiment was first performed using serum AMH, a sensitive biomarker of ovarian reserve (32). AMH is one of the most reliable indicators of ovarian reserve and reflects the functional status of growing follicles. Three extract doses (12.5, 25, and 50 mg/kg) were evaluated. Of these, only the 12.5 mg/kg dose significantly improved serum AMH levels compared with those in the DOR group (P = 0.0043). Therefore, this dose was selected for subsequent endocrine and histopathological evaluations (Figure 2A).

**Figure 2.**
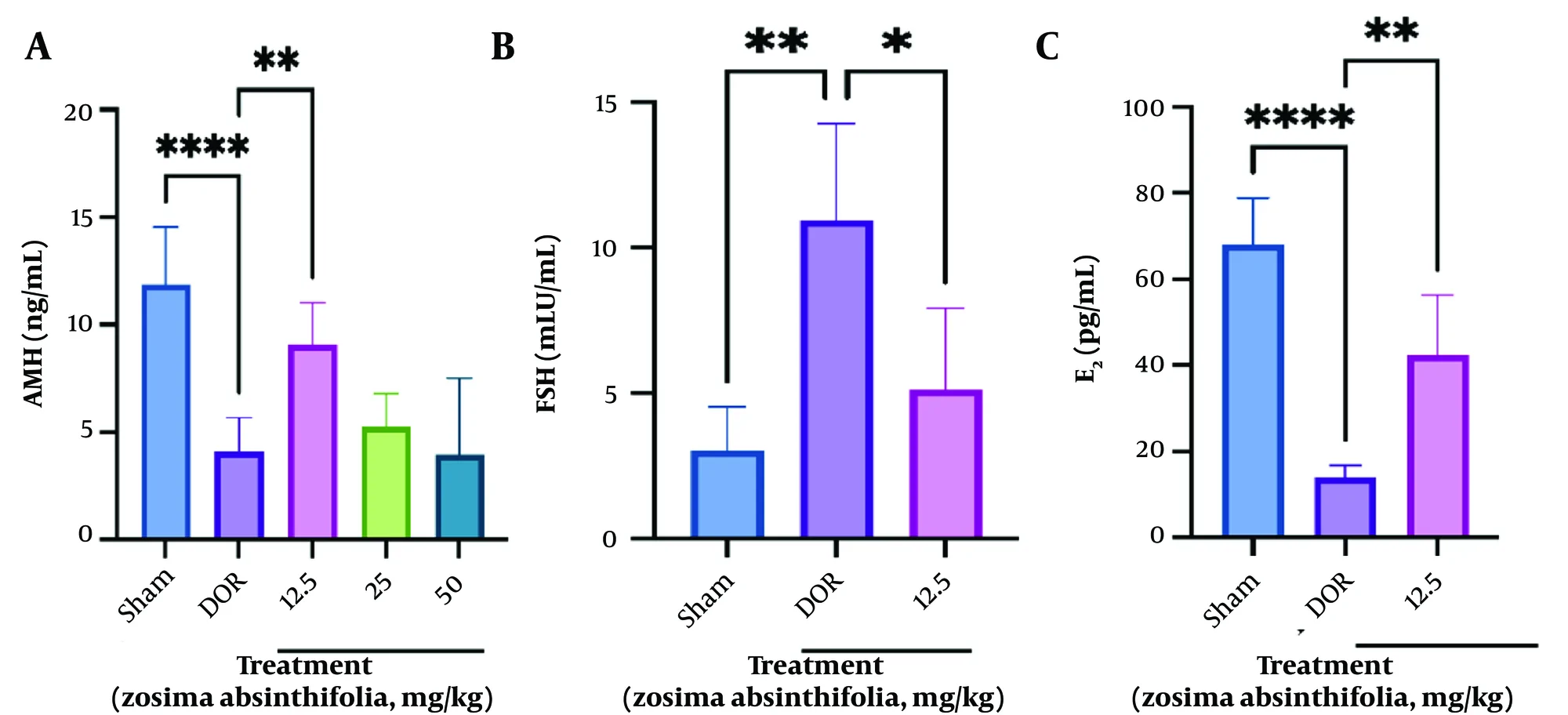
Serum levels of A) anti-Müllerian hormone (AMH) in the rat diminished ovarian reserve (DOR) model after administration of *Zosima absinthifolia* extract (12.5, 25, or 50 mg/kg) for 28 days; B) follicle-stimulating hormone (FSH); and C) estradiol (E_2_) in the rat DOR model after administration of *Zosima absinthifolia* extract (12.5 mg/kg) for 28 days. *P < 0.05, **P < 0.01, ****P < 0.0001. Values are expressed as mean ± standard deviation (SD) (n = 6).

Serum FSH levels were significantly higher in the DOR group than in the sham group (P = 0.0014). After 28 days of treatment with *Z. absinthifolia*, serum FSH levels significantly decreased compared with those in the DOR group (P = 0.0223) (Figure 2B).

Compared with the sham group, the DOR group had significantly lower E_2_ levels (P < 0.0001). Treatment with the plant extract significantly increased E_2_ levels compared with those in the DOR group (P = 0.0015) (Figure 2C). The observed improvements in AMH, FSH, and E_2_ concentrations suggest a beneficial effect of the extract on ovarian endocrine function.

In DOR rats, mild-to-moderate degeneration of ovarian follicles, a mild-to-moderate increase in stromal connective tissue, vascular congestion, and inflammatory-cell infiltration were observed (Figure 3). The characteristic ovarian findings in DOR rats included a clear decrease in antral follicles and a marked reduction in the number of growing follicles, consistent with ovarian atrophy compared with the sham group (Figure 4). In DOR rats treated with a low dose of *Z. absinthifolia* (12.5 mg/kg) for 28 days, mild-to-moderate degeneration of ovarian follicles, a mild-to-moderate increase in stromal connective tissue, vascular congestion, and inflammatory-cell infiltration were observed (Figure 5). The numbers of primordial and antral follicles were significantly lower in the DOR group than in the sham group (P = 0.0045 and P = 0.017, respectively). Administration of 12.5 mg/kg of the plant extract increased the numbers of primordial, primary, and antral follicles compared with those in the DOR group; however, the differences were not statistically significant (P > 0.05). Differences in cystic follicles, Graafian follicles, and corpora lutea among the groups were not significant (Table 2). Inflammatory status was significantly greater in the DOR group than in the sham group (P = 0.0026). Although inflammation decreased after treatment with *Z. absinthifolia* compared with that in the DOR group, the difference was not significant (P = 0.6709) (Table 2).

**Table 2.** Mean Follicle Numbers in Selected Groups ^a^

Group	Sham	DOR	*Zosima absinthifolia* (12.5 mg/kg)
**Primordial follicles**	34.8 ± 5.11	24.6 ± 2.96 ^b^	27.6 ± 3.64
**Primary follicles**	17.2 ± 4.54	12.4 ± 2.30	14.6 ± 2.96
**Secondary follicles (antral)**	3.4 ± 0.89	1.4 ± 0.89 ^c^	2.2 ± 1.09
**Cystic follicles**	1.0 ± 0.70	1.8 ± 0.44	1.4 ± 0.54
**Corpus luteum**	0.8 ± 0.44	0.8 ± 0.44	0.8 ± 0.44
**Graafian follicles**	0.2 ± 0.44	0.2 ± 0.44	0.2 ± 0.44
**Inflammatory status**	0.2 ± 0.44	1.2 ± 0.44 ^b^	1.0 ± 0.00

^a^ Values are expressed as mean ± SD. Values represent the mean of both ovaries for each rat. Follicle counts included only follicles containing a visible oocyte nucleus.

^b^ P < 0.01 relative to the sham group.

^c^ P < 0.05.

**Figure 3.**
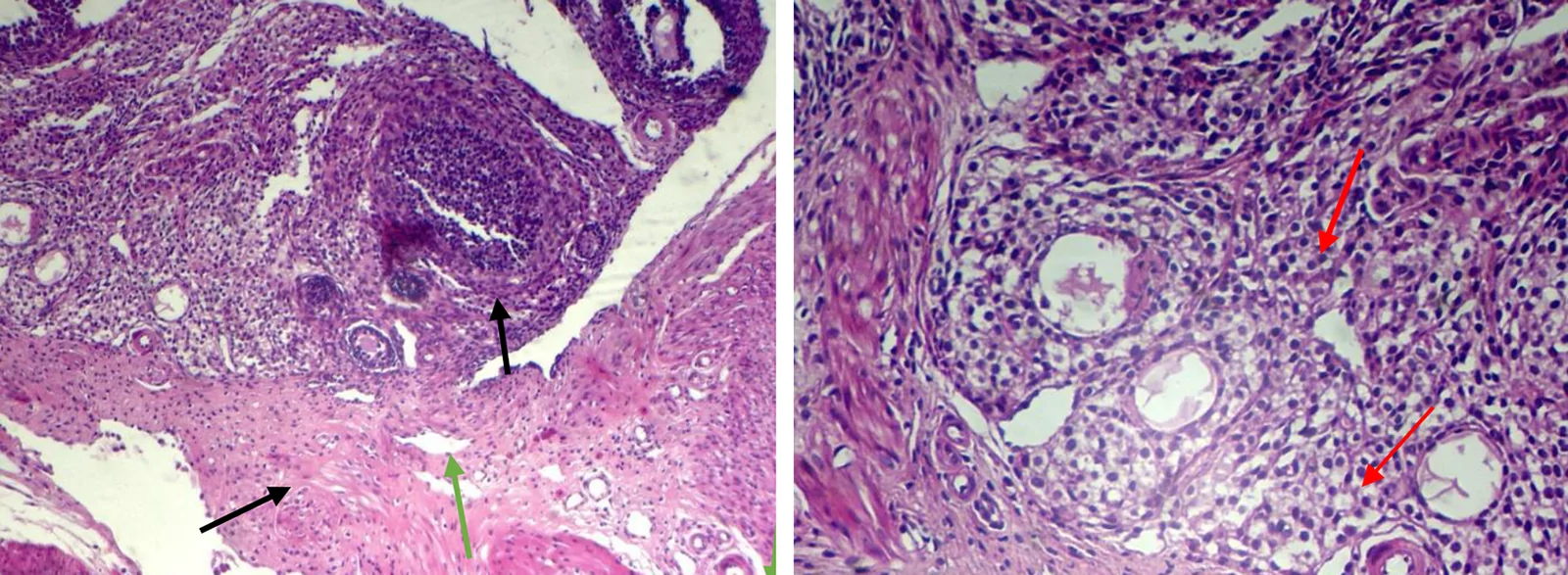
Morphological alterations in ovaries of rats with diminished ovarian reserve (DOR). Hematoxylin and eosin (H&E) staining of ovaries. Magnification ×40 and ×100 (n = 5). Red arrow: inflammatory cells; black arrow: connective tissue; green arrow: vascular congestion and obvious decrease in antral follicles.

**Figure 4.**
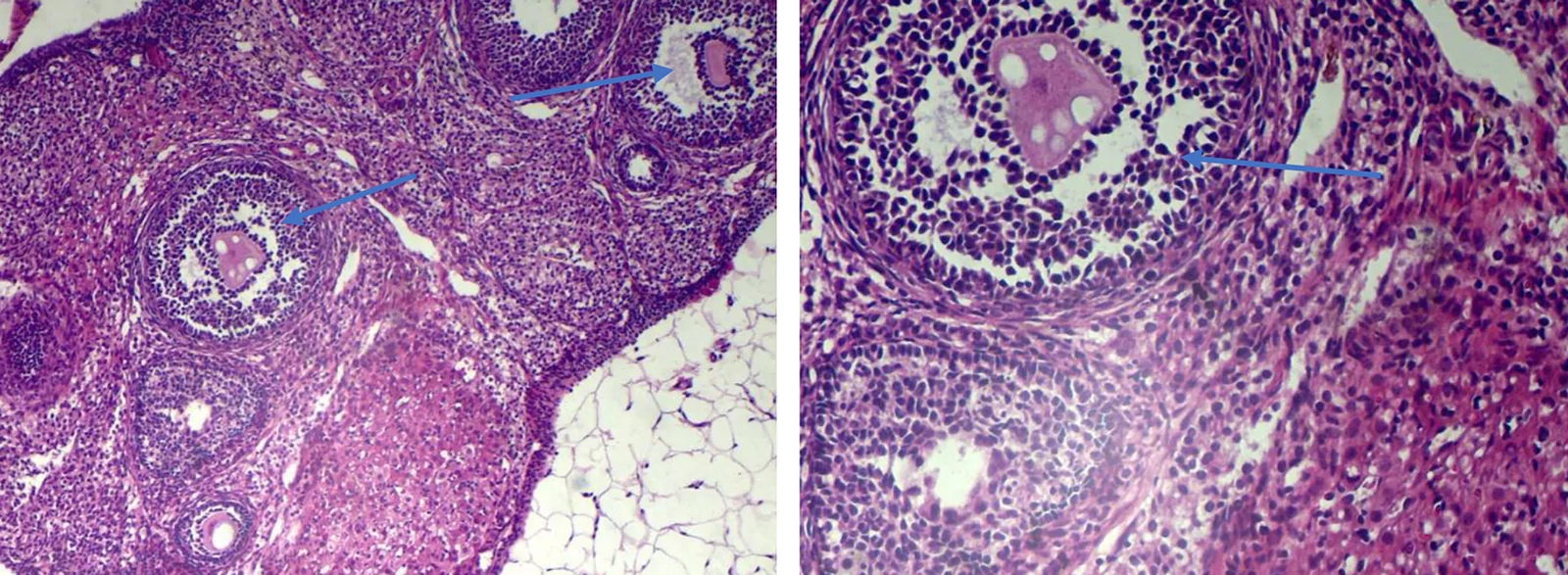
Ovarian morphology in the sham group. Hematoxylin and eosin (H&E) staining of ovaries. Normal connective tissue without inflammatory cells; blue arrow: antral follicles within the normal range. Magnification ×40 and ×100 (n = 5).

**Figure 5.**
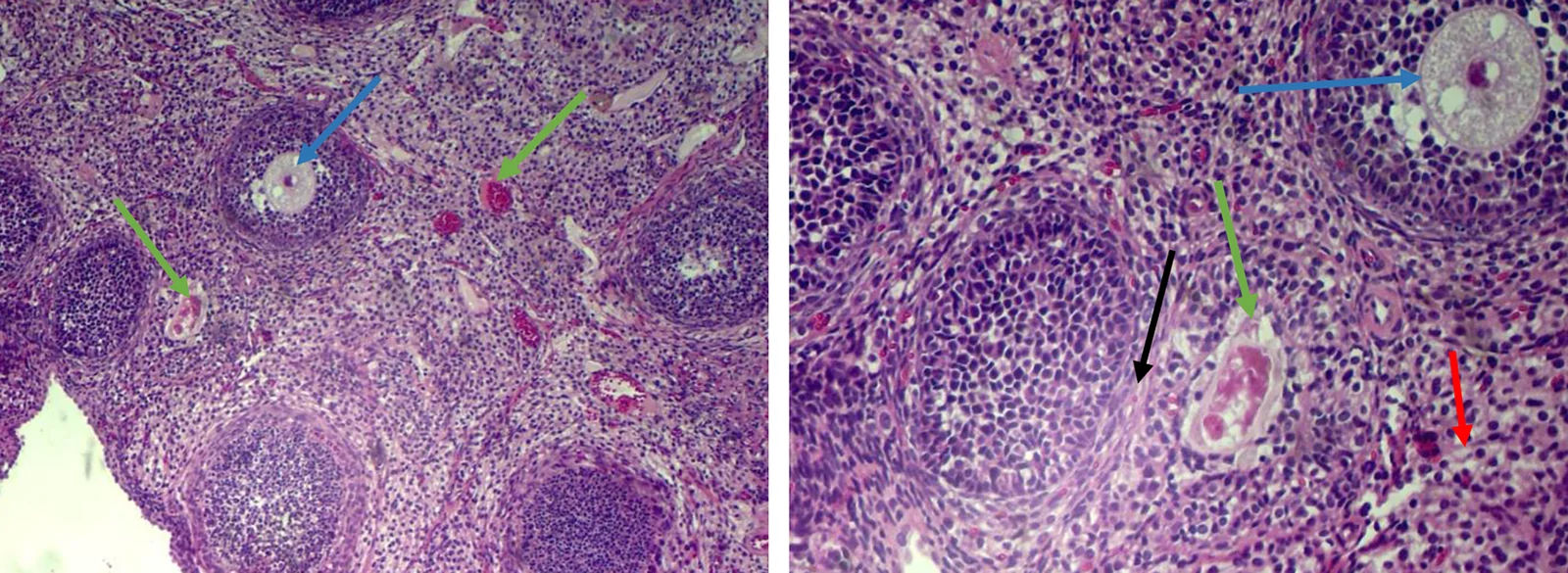
Morphological alterations in ovaries of rats with diminished ovarian reserve (DOR) treated with *Zosima absinthifolia* extract (12.5 mg/kg) for 28 days. Hematoxylin and eosin (H&E) staining of ovaries. Magnification ×40 and ×100 (n = 5). Red arrow: inflammatory cells; black arrow: connective tissue; green arrow: vascular congestion; blue arrow: antral follicles.

## 5. Discussion

### 5.1. Interpretation of Findings

In the present study, the endocrine findings provide stronger evidence of biological activity than the follicle counts. This apparent discrepancy may reflect differences in the sensitivity of functional and morphological outcome measures. Endocrine biomarkers, particularly AMH, are sensitive indicators of ovarian function and may respond earlier than structural restoration of ovarian tissue. Because AMH is secreted by granulosa cells of growing preantral and small antral follicles, improvements in hormone concentrations may indicate enhanced follicular function even in the absence of detectable changes in follicle number. Furthermore, follicle counts exhibit substantial biological variability, and the exploratory design of the present study, together with the relatively small sample size, may have limited the ability to detect statistically significant histological differences. Therefore, the endocrine and histological findings should be interpreted as complementary rather than contradictory in the assessment of ovarian function. Given the exploratory nature of the study, the findings should be interpreted cautiously, particularly because a complete dose-response relationship was not evaluated across all outcome measures. Future studies with larger sample sizes and comprehensive evaluation of all dose groups are warranted to further characterize the histological response.

Evaluation of ovarian reserve is important for predicting female fertility. Major features of DOR include reductions in the quantity and quality of oocytes, leading to decreased female fertility (28). The complex pathogenesis of DOR involves pathways such as DNA damage (33, 34), mitochondrial dysfunction (34, 35), and hormonal imbalances. Anti-Müllerian hormone deficiency reduces the inhibition of primordial follicle recruitment and accelerates follicle depletion (34). Elevated FSH and luteinizing hormone (LH) levels correlate with diminished follicular response (34). Loss of androgen receptor (AR) activity in granulosa cells triggers apoptosis (36), while lipid and glucose metabolic defects (34), apoptosis, and genetic and environmental factors also contribute (33, 37). Chemotherapeutic agents such as cyclophosphamide induce reactive oxygen species (ROS) and DNA cross-linking, thereby damaging primordial follicles (36, 37).

Cyclophosphamide is an alkylating agent that causes substantial reproductive toxicity and induces oxidative stress (38, 39). It damages the ovaries by accelerating follicle depletion, leading to DOR, and disrupts hormonal function by reducing estradiol and progesterone levels while increasing FSH and LH levels, thereby disrupting menstrual cycles (40). Accordingly, in the present study, a single 45 mg/kg dose of cyclophosphamide induced significant and consistent alterations in the estrous cycle and serum AMH levels and decreased the numbers of primordial and antral follicles, established markers of DOR. After DOR was established, treatment with the herbal extract was initiated based on the premise that plants may have potential in the management of menstrual health and DOR through antioxidant, anti-inflammatory, and anti-apoptotic activities (41-43).

*Zosima absinthifolia* is rich in beneficial compounds and has long been used for health-related and culinary purposes. It exhibits a range of reported pharmacological effects, including antidiabetic, antibacterial, antifungal, antioxidant, and anti-inflammatory activities (11, 12). It is indigenous to Iran, and despite its marked similarity to *Heracleum* species, the chemical contents of the 2 genera differ. The genus *Zosima* is enriched with pyranocoumarins, which have not been found in *Heracleum* species (44). *Zosima absinthifolia* may also be adulterated or substituted in commerce for *Levisticum officinale* W.D.J.Koch, which may result in a loss of efficacy; therefore, correct identification of this medicinal plant is important (45). Coumarins and essential oils are major components of the Apiaceae family and the *Zosima* genus; bergapten, imperatorin, pimpinellin, and umbelliferone have been isolated from the plant (20). Octyl acetate and 1-octanol in the essential oil of *Z. absinthifolia* fruits exhibited strong antimicrobial effects (20), and an in vivo study reported anti-inflammatory effects of deltoin and columbianadin from *Zosima* (46). *Zosima absinthifolia* extract is rich in phenolics with potent antioxidant activity (20, 21); anticholinesterase activity of umbelliferone has also been demonstrated (20), and umbelliferone showed aphrodisiac effects in rats with diabetes-induced erectile dysfunction (47). Bergapten and pimpinellin, which are methoxyfuranocoumarins, have shown acetylcholinesterase (AChE) and butyrylcholinesterase (BChE) inhibitory activity in vitro and in silico (48). In India, the plant is used for diabetes and in tonic formulations (49). Because DOR arises from detrimental effects on oocyte DNA, energy metabolism, and survival signaling (34), targeting these pathways with *Z. absinthifolia*, which has antioxidant and anti-inflammatory properties and potential hormone-regulatory effects, may help mitigate ovarian reserve decline.

As Frungieri et al. (50) noted, natural anti-inflammatory and antioxidant agents may help preserve ovarian health against oxidative stress, apoptosis, and inflammatory responses in gonadal tissue. In the present study, *Z. absinthifolia* had a positive effect on the DOR-related hormone profile of damaged ovaries, suggesting potential relevance to reproductive health. This effect may be attributable to bioactive constituents such as umbelliferone, which may enhance antioxidant capacity and inhibit nitric oxide production and lipid peroxidation (51), as well as phenolic compounds that act as natural antioxidants (21). In addition, coumarins such as deltoin and columbianadin have anti-inflammatory properties (46), which may contribute to potential beneficial effects on reproductive health. However, because inflammatory mediators and antioxidant activity were not directly measured in the present study, this proposed mechanism remains speculative and should be confirmed in future mechanistic studies.

Diminished ovarian reserve is a substantial challenge in reproductive medicine and often requires a multifaceted treatment approach. While conventional therapies focus on hormonal support and ovarian stimulation, an increasing body of research has investigated herbal ingredients, particularly antioxidants, as complementary approaches. Coumarins found in various medicinal plants can scavenge free radicals and modulate inflammatory pathways (46); therefore, they may be beneficial in mitigating the oxidative stress and inflammation implicated in ovarian aging and DOR. The role of antioxidants in counteracting the detrimental effects of ROS on oocytes and ovarian tissue is well established. Exogenous antioxidants, whether administered directly or derived from herbal sources rich in polyphenols, flavonoids, and vitamins, may help restore redox balance within the ovaries. This, in turn, may improve oocyte quality, enhance follicular development, and potentially preserve remaining ovarian function (39). Although research on the specific mechanisms and clinical efficacy of these natural compounds in DOR is ongoing, their integration into treatment strategies alongside established medical interventions may have potential to improve outcomes in women with DOR.

The protective effects observed in the present study cannot be attributed solely to coumarins. The total extract of *Z. absinthifolia* contains a diverse array of phytochemicals, including coumarins, phenolic compounds, flavonoids, and other secondary metabolites (11), which may contribute individually or synergistically to the observed biological effects. Because the present investigation evaluated the crude extract rather than isolated constituents, the specific compounds responsible for the observed ovarian-protective effects remain to be identified through bioactivity-guided fractionation and mechanistic studies. Further studies using HPLC- or LC-MS-based phytochemical profiling and identification of marker compounds are also needed to achieve more comprehensive standardization.

Use of herbal ingredients requires oversight by health care professionals. Rigorous clinical trials are needed to establish the safety, optimal dosages, and efficacy of coumarins and other antioxidants in the management of DOR.

A challenge with plant extracts is that low doses may have therapeutic effects, whereas higher doses may produce different or opposite pharmacological effects. In the present study, as in a previous study of clove extract in DOR (52), higher doses of clove and *Z. absinthifolia* did not significantly improve AMH levels. One possible explanation is that increasing the extract dose increases the concentrations of compounds with antagonistic effects, thereby masking the positive effects of other substances with agonistic properties.

Collectively, the present findings provide preliminary evidence supporting the biological activity of *Z. absinthifolia* in this experimental model.

### 5.2. Study Limitations

The cost of hormone measurements constrained the animal sample size. In addition, the study did not assess immunological or oxidative-stress parameters, including inflammatory factors such as interleukins. Further mechanistic, toxicological, pharmacokinetic, and clinical studies are required before the potential therapeutic value of *Z. absinthifolia* can be established.

### 5.3. Conclusions

The present findings provide preliminary evidence that *Z. absinthifolia* may preserve ovarian function in a cyclophosphamide-induced rat model of DOR. Although endocrine markers of ovarian reserve improved significantly, histopathological findings were less pronounced, and follicle counts did not show statistically significant recovery. Therefore, these findings should be interpreted as evidence of potential ovarian protection rather than definitive restoration of ovarian reserve. Further studies investigating toxicity, reproductive safety, dose-response relationships, and controlled clinical trials are required before potential clinical application can be considered.

## Data Availability

The data is available on request from the corresponding author during submission or after publication.
